# A Systematic Review of Telehealth Applications in Endocrinology

**DOI:** 10.1089/tmr.2024.0032

**Published:** 2024-09-27

**Authors:** SeyedAhmad SeyedAlinaghi, Soudabeh Yarmohammadi, Pegah Mirzapour, Soheil Dehghani, Sepide Ahmadi, Faeze Abbaspour, Ava Pashaei, Ayoob Molla, Alireza Pashaei, Samin Ahmadi, Esmaeil Mehraeen

**Affiliations:** ^1^ Iranian Research Center for HIV/AIDS, Iranian Institute for Reduction of High Risk Behaviors, Tehran University of Medical Sciences, Tehran, Iran.; ^2^ Research Development Center, Arash Women Hospital, Tehran University of Medical Sciences, Tehran, Iran.; ^3^ Trauma Research Center, Kashan University of Medical Sciences, Kashan, Iran.; ^4^ School of Medicine, Tehran University of Medical Sciences, Tehran, Iran.; ^5^ School of Nursing, University of British Columbia, Vancouver, British Columbia, Canada.; ^6^ School of Medicine, Bushehr University of Medical Sciences, Bushehr, Iran.; ^7^ Faculty of Nursing, Department of surgical technology, Zanjan University of Medical Sciences, Zanjan, Iran.; ^8^ Department of Health Information Technology, Khalkhal University of Medical Sciences, Khalkhal, Iran.

**Keywords:** telehealth, telemedicine, endocrinology, health care

## Abstract

**Introduction:**

The prevalence of telehealth has witnessed a significant increase in various medical domains, especially in endocrinology. Telehealth brings about considerable advantages for both patients and health care professionals. However, despite these positive aspects, the growing prominence of telehealth is accompanied by certain challenges. This systematic review aims to assess the role of telehealth in endocrinology, including its applications, effectiveness, challenges, and implications for patient care.

**Methods:**

This study involved a thorough search using comprehensive techniques across databases such as PubMed/Medline, Embase, and Scopus. The studies were selected for a tailored adaptation of the Preferred Reporting Items for Systematic Reviews and Meta-Analyses (PRISMA) to enhance the clarity of our systematic review’s reporting.

**Results:**

This systematic review explores global telemedicine applications in endocrinology. Addressing various endocrine conditions, interventions utilize technology tools such as smartphones and applications, offering multifaceted utility from education and data gathering to screening and treatment. Notably, these interventions demonstrate adaptability during the COVID-19 pandemic. Positive outcomes include enhanced patient education, disease self-management, reduced complications, and improved glycemic control. However, drawbacks include the need for technical proficiency, perceived lower care quality, and potential privacy risks. These nuanced findings contribute to the discourse on telemedicine efficacy and limitations.

**Conclusion:**

In conclusion, telehealth holds significant potential in transforming endocrine care. While there are challenges to its implementation, the benefits it offers underscore its value as a health care delivery model.

## Introduction

Recently, the application of new technologies in disease management has increased.^1–4^ Telehealth involves utilizing digital communication technologies to deliver health care services remotely.^5^ It extends beyond patient care, also serving as a platform for disseminating information and providing education in the health care domain.^6^ Endocrinology is a particularly well-suited field for telehealth because its focus is on data monitoring, chronic condition management, and lifestyle counseling, and the assessment of diverse endocrine conditions is mainly reliant on laboratory analyses, or imaging outcomes, rather than physical examination findings.^7,8^ Following the initial phases of the COVID-19 pandemic, during which telehealth saw a surge in popularity among patients, endocrinology stood out as one of the subspecialties experiencing a notable rise in telehealth visits.^9,10^ Given that diabetes ranks among the most prevalent endocrine disorders, imposing a considerable burden on patients, telehealth has emerged as a widely utilized tool in this realm.^11,12^ Its application is particularly prominent in diabetes care, notably in blood sugar control, diabetes management, and screening for diabetes.^13,14^ The increasing cost of health care and the need for enhanced treatment options are prompting more hospitals to explore the advantages of telemedicine.^15^ Telehealth mitigates the workload for doctors and contributes to increased job satisfaction.^16^ These facilitate the smooth sharing of information, ensuring the appointments occur on time. Telehealth offers advantages for patients by higher completion rates of visits and a reduction in the travel burden for patients, including reminders for medications and appointments.^5^

Telehealth faces challenges related to factors such as ethnicity, age, language barriers, and limited access to technology.^17,18^ Hence, the drawbacks of telehealth, including cost implications, restricted accessibility to reliable internet connections and technology devices, privacy concerns as individuals may worry about the security of their personal health information, and reduced opportunities for physical interactions which potentially impact the quality of the patient–provider relationship, should be duly considered.^19^

In this systematic review, the authors aimed to comprehensively evaluate the role of telehealth in the field of endocrinology, examining its applications, effectiveness, challenges, and implications for patient care.

## Methods

To explore the landscape of telehealth in endocrinology, we chose a systematic approach. This involved a thorough search using comprehensive techniques across databases such as PubMed/Medline, Embase, and Scopus. The studies were selected for a tailored adaptation of the Preferred Reporting Items for Systematic Reviews and Meta-Analyses (PRISMA) to enhance the clarity of our systematic review’s reporting.

### Search strategy

In collaboration with a research librarian, we carefully devised a search strategy targeting PubMed/Medline, Embase, and Scopus databases. Our key search terms were “telemedicine,” “diabetes,” and “endocrinology” Using various search functions, such as thesauruses, Boolean operators, truncation, and advanced searches, we conducted this thorough search on June 23, 2024. There were no restrictions on publication date or country of origin; however, our searches focused on studies of adults published in English (Appendix 1). Additionally, we expanded our search by reviewing the reference lists of the identified articles to find more probable relevant publications.

### Study selection

The references were imported into Rayyan, an online systematic review tool, following their download into Endnote. Initially, two independent authors reviewed titles and abstracts based on inclusion criteria, with any inclusion-related questions referred to a third author for resolution. The third author independently examined a subset (5%) of study titles and abstracts for eligibility to ensure consistent adherence to inclusion criteria. All studies deemed for inclusion by at least one investigator underwent a full-text review. Studies marked for exclusion at the title and abstract level by one investigator were screened by a second investigator. If both agreed on exclusion, the study was excluded. The full-text review involved two independent reviewers, and conflicts were resolved through discussion.

Study eligibility criteria, including population, intervention, comparator, outcome, timing, setting elements, and additional criteria such as study design, language, and publication type, were organized. Four inclusion criteria guided literature selection: (1) the study had to be original; (2) the exposure included empirical data on adherence to data logging processes using advanced technology for adults with endocrinology components; (3) the outcome focused on the impact of telehealth in endocrinology; (4) risk estimates with 95% confidence intervals. Ultimately, among the publications, we identified a total of 40 studies deemed applicable. Excluded were conference abstracts, unpublished studies (dissertations and theses), editorials, opinions, and discussion papers.

### Definition of terms

We identified key elements to abstract from eligible literature to gather essential evidence on conducting telehealth visits across various clinic settings. Building on this foundation, we examined the relationship between clinical visits and telehealth modalities (e.g., telephone, video, and in-person) outcomes. Our telehealth interventions aligned with a well-defined telehealth concept, encompassing crucial contextual factors such as delivery mode (telephone, video, and in-person), dose (duration and frequency of contact), and the clinical context of care provision. Moreover, we specified that telehealth-delivered care should pertain to clinical activities conducted by the prescribing clinician, such as evaluation, diagnosis, or medication prescription. It excluded interventions such as self-management education or other support provided adjunctively by a clinical team member other than the prescribing clinician (e.g., nurse care manager), as these have been previously evaluated.

### Quality and risk of bias assessment

To optimize the quality, this review study benefits from the Preferred Reporting Items for Systematic Reviews and Meta-Analyses (PRISMA) checklist. To minimize any probable bias risk, we utilized the Newcastle-Ottawa Scale (NOS) risk assessment tool (Table 1). Worthy to mention that a total score of nine in three categories is calculated in this numerical bias assessment tool. These three categories include selection, comparability, and exposure/outcome. Numerical values of four, two, and three are attributed to these categories respectively.

**Table 1.** Newcastle-Ottawa Scale Bias Risk Assessment of the Study

ID	Selection (out of 4)	Comparability (out of 2)	Exposure/Outcome (out of 3)	Total (Out of 9)
1	4	2	3	9
2	4	2	3	9
3	2	1	2	5
4	4	1	3	8
5	3	2	2	7
6	3	2	3	8
7	3	2	3	8
8	2	1	2	5
9	3	2	3	8
10	4	1	3	8
11	3	2	2	7
12	4	2	3	9
13	4	2	3	9
14	2	1	2	5
15	3	2	3	8
16	3	2	3	8
17	3	2	3	8
18	2	1	2	5
19	2	0	3	5
20	2	1	1	4
21	3	1	3	7
22	2	1	2	5
23	4	2	2	8
24	2	1	3	6
25	2	0	3	5
26	2	1	2	5
27	4	2	3	9
28	2	1	2	5
29	4	2	2	8
30	2	1	2	5
31	4	2	3	9
32	2	1	3	6
33	2	0	3	5
34	2	1	1	4
35	3	1	3	7
36	4	2	3	9
37	4	2	2	8
38	3	2	2	7
39	3	2	3	8
40	4	2	3	9
41	4	2	2	8
42	3	2	3	8

**Good quality: **3 or 4 stars in the selection domain AND 1 or 2 stars in the comparability domain AND 2 or 3 stars in the exposure/outcome domain.

**Fair quality: **2 stars in the selection domain AND 1 or 2 stars in the comparability domain AND 2 or 3 stars in exposure/outcome domain.

**Poor quality: **0 or 1 star in selection domain OR 0 stars in comparability domain OR 0 or 1 stars in exposure/outcome domain.

### Data extraction and synthesis

We presented a comprehensive summary of the primary literature, extracting relevant data from eligible studies. Summary tables delineate key characteristics, including study design, patient demographics, and details of the intervention and comparator. Due to conceptual heterogeneity in the structure, purpose, and delivery of telehealth visits, we opted not to conduct a meta-analysis. Instead, we provided a narrative description, emphasizing the identification of patterns in the efficacy and safety of interventions across conditions and outcome categories. Continuous outcomes were synthesized using the mean patient-level difference (follow-up minus baseline) when reported on the same scale. For studies without direct reporting of mean and standard deviation (SD) of patient differences, we calculated the difference in means between follow-up and baseline. If only baseline SD were reported, we assumed the same SD at follow-up. Without other information, a conservative 0.5 correlation between follow-up and baseline measures was assumed.

## Results

### Overview of included articles

**FIG. 1.**
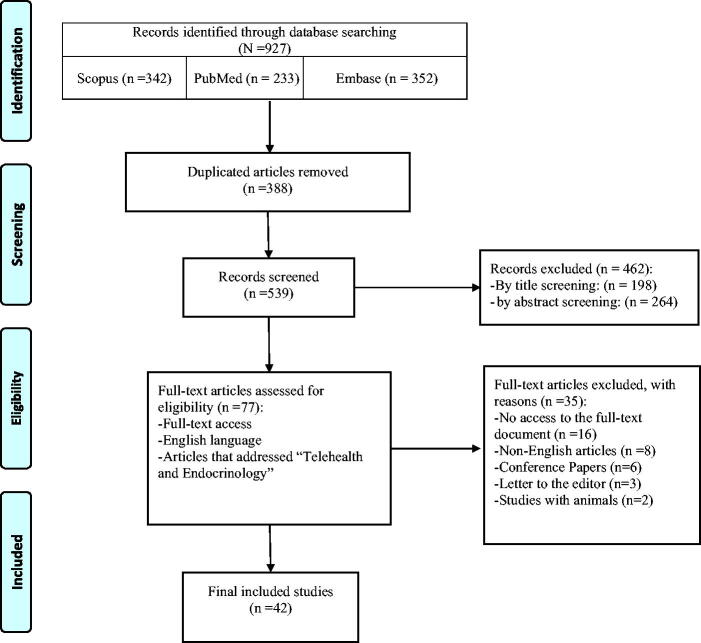
Telehealth and endocrinology: A systematic review.

This systematic review comprehensively examines global telemedicine applications in endocrinology, covering studies conducted between 2004^20^ and 2024,^21,22^ with more than half of the articles being published post-2018. We included 42 articles for full-text review (Fig. 1). As shown in Table 1, according to the NOS risk assessment tool, out of 42 included articles, 5 articles were of good quality (≥6), 13 articles were of fair quality (5≥, >2), and none of the articles were of poor quality (2≥).

The research, involving over 25,000 participants, spans diverse countries, with notable contributions from the United States of America,^23–34^ Pakistan,^35–37^ Turkey,^38,39^ India,^40,41^ Korea,^20,42^ Australia,^43,44^ Saudi Arabia,^45,46^ China,^21^ and Iran.^22,47^ We reviewed incorporating varied study types, such as randomized controlled trials,^32,35,36,38,41,44,48–52^ observational studies such as cohorts,^20,23,24,29–31,45,53–55^ case-controls,^56^ and cross-sectional studies.^25,26,33,46,47,57,58^ The mean age of participants varied from less than one year^54^ to 68 years,^53^ demonstrating the wide applicability of telemedicine across various age demographics. In terms of gender distribution, most studies included both male and female participants. However, a study focusing on telehealth visits for patients dealing with reproductive endocrinology and infertility issues was an exception, as it exclusively featured female participants^25^ (Table 2).

**Table 2.** Details of the Included Articles

ID	Year and reference	Type of study	Target group	Sample size (female/male)	Gender	Age	Country
1	(2017)^35^	RCT	Patients with diabetes	*n* = 2,265	Male: 1,117 (49.3%) Female: 1,148 (50.7%)	48.76 ± 14.25	Pakistan
2	(2020)^45^	Cohort study	Patients with diabetes	*n* = 145 Video conferencing: 100 Audio conferencing: 45	Male: 47 (32%) Female: 98 (68%)	N/A	Saudi Arabia
3	(2023)^23^	Cohort study	Patients with diabetes	*n* = 234	Male : 111 (47%) Female: 123 (53%)	47 ± 14	USA
4	(2020)^24^	Cohort study	Patients with endocrine disease	*n* = 834 Two groups: 1) Medicaid patients referred to an endocrinologist in the year prior to eConsult implementation: 365 2) Those referred in the 1 year after implementation: 469–229 of the 469 endocrinology referrals (49%) received an eConsult whereas 240 patients were referred directly for face-to-face visits.	1) Medicaid patients referred to an endocrinologist in the year prior to eConsult implementation: Male: 106 (29%) Female: 258 (70.7%) 2) Those referred in the 1 year after implementation: Male: 122 (26.4%) Female: 345 (73.6%)	Pre-eConsults = (mean: 41), post-eConsults = (mean: 43.8)	USA
5	(2022)^25^	Cross-sectional study	REI patients	*n* = 101	Female: 101	32.8 ± 5.2	USA
6	(2023)^26^	Cross-sectional study	Endocrinologists	*n* = 200 notes of 10 endocrinologists (10 IP and 10 telehealth visits)	N/A	N/A	USA
7	(2006)^53^	Cohort study	Patients with diabetes	Treatment group: 391 Comparison group: 391	N/A	Mean: 68.1	USA
8	(2012)^48^	RCT	Patients with poorly controlled type 1 or type 2 diabetes (A1C >8.0%)	*n* = 65 Video messages group: 31 Usual care group: 33	Total male: 35 (55%) Female: 30 (45%) Video messages group Male: 15 (48%) Female: 16 (52%) Usual care group Male: 20 (61%) Female: 13 (39%)	Total: 58 ± 11 Video messages group: 55 ± 10 Usual care group: 60 ± 11	USA
9	(2021)^57^	Cross-sectional study	Outpatients with endocrine diseases.	Teleendocrinology: 622 F2F: 269	N/A	N/A	Italy
10	(2021)^38^	RCT	Children with endocrine disease	*n* = 267	N/A	N/A	Turkey
11	(2020)^49^	RCT	Type 2 diabetes	*n* = 740	Male: 272 (37%) Female: 468 (37%)	53.8 ± 8.8	USA
12	(2019)^39^	RCT	Type 1 diabetes	*n* = 82	Male: 39 (47.6%) Female: 43 (52.4%)	10.89 ± 4	Turkey
13	(2022)^58^	Cross-sectional study	Type 2 diabetes	*n* = 720	Male and female (M/F ratio: 0.71)	62.5 ± 11.3	Morocco
14	(2021)^59^	Cohort study	Type 2 diabetes	*n* = 74 divided in 3 groups: Endocrine (*n* = 29), PharmD (*n* = 32), e-consult (selected by clinical pharmacists to have a CGM-enhanced eConsult) (*n* = 13)	Total Male: 40 (54.1%) Female: 34 (45.9%) Endocrine Male: 14 (48.3%) Female: 15 (51.7%) PharmD Male: 26 (57.8%) Female: 19 (42.2%)	53.9 + 9.2	USA
15	(2013)^43^	RCT	Patients with diabetes	*n* = 56	Male: 31 (55%) Female: 25 (45%)	Mean: 51	Australia
16	(2014)^44^	RCT	Patients with diabetes	*n* = 160 reference group = 80 (received 2 face-to-face consultation), telemedicine group = 80 (*n* = 40 received first face-to-face then videoconference consultation, *n* = 40 received first videoconference then face-to-face consultation)	N/A	N/A	Australia
17	(2019)^27^	Cohort (prospective) study	Patients with high-rated DR^*^ and aged-related macular degeneration	*n* = 159	F/M ratio: 1.3	65 ± 17	USA
18	(2021)^17^	Cohort study	Patients with diabetes	*n* = 552	Male: 263 (47.6%) Females: 289 (52.4%)	1–24: (*n* = 189), 25–49: (*n* = 91), 50–65: (*n* = 138), and >65: (*n* = 126)	USA
19	(2023)^54^	Cohort study	Pediatric patients receiving endocrinology specialty care	*n* = 5,083	Male: 2,396 (47.1%) Females: 2,687 (52.9%)	Adolescents-aged: 13–17 (36.6%), School-aged: 7–12 (36.6%), Preschool-aged: 1–6 (20.7%), and Less than 1 year of age: 6.2%	USA
20	(2021)^56^	Case-control study	Type 2 diabetes	*n* = 30 (intervention group: 15, control group: 15)	Intervention group Male: 6 Females: 9 Control group Male: 5 Female: 10	50.9 ± 11.8	Iceland
21	(2021)^50^	RCT	Adolescents with type 1 diabetes and their parents	*n* = 25	Male: 13 (52%) Females: 12 (48%)	12.28 ± 1.62	USA
22	(2017)^46^	Cross-sectional study	Patients with diabetes	*n* = 1,024 [2,048 eyes]	N/A	50.62 ± 10.13	United Arab Emirates
23	(2008)^42^	RCT	Patients with diabetes	Intervention group = 18 Control group = 16	Intervention group Male: 9 (50%) Females: 9 (50%) Control group Male: 7 (43.8%) Female: 9 (56.2%)	Intervention group: 45.5 ± 9.1, control group: 48.5 ± 8.0	Korea
24	(2018)^51^	RCT	Type 1 diabetes	Intervention group *n* = 48 Control group *n* = 46	Intervention group Male: 25 (52.08%) Females: 23 (47.92%) Control group Male: 22 (47.83%) Female: 24 (52.17%)	Intervention group: 26.35 ± 7.36, Control group: 27.63 ± 7.25	Greece
25	(2004)^20^	Cohort study	Patients with diabetes	*n* = 185	Male =132 Female = 53	42.4 (8–79)	Korea
26	(2006)^60^	Cohort study	Patients with type 1 diabetes	*n* = 13 newly diagnosed type 1 diabetic patient	Male: 8 Female: 5	25.6 ± 6.3	Poland
27	(2016)^29^	Cohort study	Veterans with diabetes mellitus	*n* = 189 Face-to-face consultation (*n* = 85) Teleconsultation (*n* = 104)	Teleconsultation Male: 96 (92.3%) Females: 8 (7.7%) Face-to-face consultation Male: 83 (97.6%) Female: 2 (2.4%)	Teleconsultation: 63.6 ± 10.3 Face-to-face consultation: 61.7 ± 9.8	USA
28	(2022)^55^	Cohort study	Obese patients	*n* = 202 First-time teleconsultation (*n* = 88) Follow-up consultation (*n* = 114)	N/A	First-time teleconsultation: 39 (32–45) Follow-up consultation: 41 (33–52)	Colombia
29	(2020)^61^	Cohort study	Pediatric with type 1 diabetes	*n* = 64	N/A	N/A	Canada
30	(2018)^30^	Retrospective study	DR	*n* = 10,223	N/A	N/A	USA
31	(2022)^31^	Cluster-randomized matched cohort study	Type 2 diabetes	*n* = 260	Control group= female: 58 (44.6), male: 72 (55.4%)-Intervention group = female: 61 (46.9%), male: 69 (11.6%)	Mean: control group = 61.3, Intervention group = 62.6	USA
32	(2014)^40^	RCT	Patients with diabetes	*n* = 100	Female: 35 (35%), male: 65 (65%)	54 + 11.5	India
33	(2015)^32^	RCT	Patients with diabetes	*n* = 100	Control group = Female: 17 (34%), Male: 66 (33%)-Intervention group = Female: 18 (36%), Male: 32 (64%)	Mean: control group = 5.6 + 10, Intervention group = 5.2 + 12	USA
34	(2022)^41^	RCT	Type 2 diabetes	*n* = 66	Female: 22 (33.33%), Male: 44 (66.66%)	42.88 ± 9.5	India
35	(2022)^33^	Cross-sectional mixed-methods study	Pediatric	*n* = 40	Female = 31 (77.5%), Male: 9 (22.5%)	13.7 ± 4.0	USA
36	(2020)^47^	Cross-sectional study	Type 2 diabetes	*n* = 176	N/A	53.18 ± 15.05	Iran
37	(2015)^36^	RCT	Patients with diabetes	*n* = 440	Control group = Female: 85 (38.6%), Male: 135 (61.4%)-Intervention group = Female: 85 (38.6%), Male: 135 (61.4%)	Control group = 49.21 ± 7.92, Intervention group = 48.95 ± 8.83	Pakistan
38	(2020)^37^	RCT	Patients with diabetes	*n* = 648	Non-SMS = Female: 72 (52%), Male: 67 (48%), SMS group: Female: 86 (45.7%), Male: 102 (54.3%)	Non-SMS: 47.06 ± 14.83, SMS group: 45.41 ± 15.13	Pakistan
39	(2018)^34^	Retrospective	Type 1 diabetes	*n* = 32	Female: 9.4%, Male: 90.6%	Mean: 53.5	USA
40	(2016)^52^	RCT	Patients with diabetes	*n* = 100	Control group = Female: 20, Male: 30-Intervention group = Female: 23, Male: 27	Control group = 53.5 ± 12.4 (18–74), Intervention group = 55.0 ± 13.1 (18–74)	China
41	(2024)^21^	Retrospective	Patients with diabetes	*n* = 93	Male: 51 (54.83%) Female: 42 (45.16%)	N/A	China
42	(2024)^22^	Retrospective	Patients with diabetes	*n* = 1,159	Male: 573 (49.43%) Female: 586 (50.57%)	Mean: 55.25	Iran

RCT, Randomized Controlled Trial.

### Telemedicine interventions and target groups

The majority of the studies primarily focus on patients as the target group for telemedicine interventions. However, one study targets endocrinologists and compares their performance in virtual visits versus in-person visits.^26^ Telemedicine interventions have been applied to a wide array of endocrine conditions. The majority of these interventions were targeted toward Type 1 and Type 2 diabetes mellitus.^17,20,23,34,35,39,41,44,45,47,48,51,52,60^ Other areas of focus included pediatric endocrinology,^33,38,54,61^ obesity,^55^ reproductive and fertility complications,^25^ and thyroid disorders.^24,57^ Additionally, telemedicine was utilized for screening conditions, such as diabetic retinopathy.^27,30,46^ The interventions employed a variety of technology tools. Smartphones^17,29,32,36,42,45,47,60^ and mobile applications^41,45,49,50,52,56,61^ were frequently used, along with traditional communication methods such as phone calls^23,35,40,51,57,58^ and emails.^23,38,51,57,61^ Other tools included SMS,^20,37,39,40,53^ video/audio visits,^26,33,43,44,48,49,55^ social messengers such as WhatsApp,^39,58^ and web-based^24,59^ platforms. In certain cases, specialized equipment such as teleophthalmological cameras and devices were also utilized.^27,30,46^ Telemedicine interventions demonstrated a multifaceted utility, serving various purposes. These ranged from education^29,32,34–38,40,52^ and data gathering^17,24–26,36,39,42,47,54,61^ to screening^27,30,46^ and treatment.^20,23,32,38,43–45,48,49,51,53,55–60^ Notably, these interventions provided enhanced support for chronic conditions, particularly during viral pandemics (Table 3).

**Table 3.** Description of the Findings Reported in Included Studies

ID	The purpose of the study	Type of disease	Type of technology	Type of intervention	The benefits of intervention/technology	Disadvantages of intervention/technology	Other findings
1	To provide continuous support for diabetes self-management, reducing acute complications, and hospitalizations.	Diabetes	Phone calls	Disseminating information and instructing patients on the self-administration of diabetes.	The study reveals that technology-driven personalized support via a helpline can avert diabetes emergencies, improving health outcomes.	N/a	The received calls were for hypoglycemia, hyperglycemia, insulin dose adjustment, the learning of insulin techniques, information on insulin handling, oral medication inquiry, diet inquiry, timings of insulin, diabetic foot-related issues, etc.
2	To regulate their blood sugar, the study focuses on ensuring uninterrupted support for diabetic patients during the pandemic via virtual visits, incorporating Google Forms for patient inquiries.	Diabetes	Diabetes telemedicine clinic protocol: a smartphone/computer with audio and video capabilities, A web-based videoconferencing software (Zoom, FaceTime, or audio-only visits), Diabetes software (by linking their devices and data to the clinic cloud-based accounts), Email/WhatsApp, Patient Request in Google Forms, Virtual Visits, a home delivery service to deliver all medications and diabetes supplies to patients, educational sessions	Sustaining communication between individuals with diabetes and their health care providers during pandemics, with the aim of ensuring uninterrupted support.	The benefits included a high level of patient satisfaction with telemedicine, as expressed by patients reporting reduced infection risk (74.4%), shorter waiting times for health care provider consultations (72.4%), eliminating the need for clinic visits (64.8%), experiencing an equivalent quality of care compared to traditional clinics (64.8%), and achieving cost savings (16.5%).	Disadvantages of telemedicine protocol reported by the patients: the need for technical proficiency (13.1%), concerns about lower care quality compared to traditional clinics (6.9%), potential privacy risks (2.7%), and perceived higher costs of telemedicine compared to in-person clinic visits (0.7%).	The study confirmed high satisfaction with the diabetes telemedicine clinic and the desire to continue post-COVID-19, resulting in reduced in-person visits without compromising care quality or satisfaction.
3	Enable holistic care and aid in the integration of continuous glucose monitoring into self-managing diabetes.	Diabetes	Virtual endocrinology clinic (video training sessions, texts, emails, and telephone calls).	The study aims to assess a virtual clinic for comprehensive diabetes care, CGM^*^ integration, and diabetes-related behavioral support.	In individuals with type 1 diabetes, mean HbA1c reduced from 7.8% to 7.1% at 6 months, with an 11% increase in TIR^*^. For type 2 diabetes, mean HbA1c dropped from 8.1% to 7.1% at 6 months, with an 18% TIR increase. Hypoglycemia was infrequent in type 2 diabetes (0.5% <70 mg/dL and 0.07% <54 mg/dL over 6 months).	N/A	This virtual model enhances access to diabetes care, particularly for those with limited in-person access. The study shows the success of diabetes telehealth services during the pandemic, underscoring their ongoing value.
4	Accessing to Endocrinology Care for Medicaid Patients	Endocrinology issues	Electronic consultations (eConsults) /a web-based eConsult platform	This study assessed eConsults’ impact on referral outcomes, time to resolution, diagnoses, and provider satisfaction.	Before eConsults, 37.8% of endocrinology referrals were completed, while after eConsults, 59.9% were completed. 41.4% of the postimplementation completions were eConsults. In general, eConsults received an 89% satisfaction rate from 32 out of 36 PCPs^*^.	19 out of 36 (53%) patients felt that the process did not create additional work or burden for them.	The broad implementation of eConsults presents a potential solution to current health care system challenges. Thyroid conditions were the most common reason for a consult
5	To assess the utility, usability, interface quality, interaction quality, reliability, and satisfaction regarding telehealth visits, along with their potential for future use.	Infertility or noninfertility indications such as recurrent pregnancy loss, PCOS^*^, or Mullerian anomalies.	Telehealth visits	The study aims to assess patient satisfaction in new patient telehealth visits in a REI* clinic. (data gathering)	The intervention brought significant benefits, with 93% patient satisfaction, improved health care access for 88%, and 96% time-saving. Interface and interaction quality received high ratings, with 93% finding it pleasant and 84% liking it. Reliability was similar to in-person visits for 68% of respondents, and 93% expressed willingness to use telehealth again	3% of patients with negative perceptions. Additionally, 16% found the system fell short of their expectations, and 4% were unconvinced about telehealth’s acceptability. Some patients preferred in-person follow-up visits (18%), and 3% would not use telehealth again, suggesting it may not be suitable for all patients.	Amid COVID-19, health care delivery transformed with a surge in telehealth. Our survey revealed high patient satisfaction, with travel time savings and improved REI care access. Notably, patient preference for in-person or telehealth did not differ based on distance, indicating overall satisfaction beyond location considerations.
6	This study addresses the problem of unclear guidance on conducting virtual PE* for diabetes in telehealth. We compare how endocrinologists document PE* components during IP* and TH visits to improve diabetes care in telehealth.	Diabetes	Video telehealth	To compare endocrinologists’ documentation of PE^*^ components for diabetes in both IP^*^ and TH^*^ visits.	N/A	This study highlights significant drawbacks in telehealth physical exams. A review of 200 visits across 10 clinicians showed much less documentation of physical exam components in telehealth for new diabetic patients compared to in-person visits. The study underscores the need for improvements in virtual physical examinations.	N/A
7	The program aims to address the issue of costly interventions, such as hospitalizations or emergency department visits, by employing nurse care coordinators.	Diabetes	A messaging device	Patients answered daily scripted questions about diabetes symptoms using a messaging device. Nurse care coordinators, following disease management protocols, prevented costly interventions by educating patients. Coordinators monitored responses daily, making clinical judgments for necessary telephone calls or appointments. Calls lasted 15–30 min on average, addressing various issues. Coordinators performed tasks like patient assessments, medication management, and appointment scheduling. In rare cases, telemonitor and videophone with 2-way audio/video connectivity were used for weekly contact.	The program significantly reduced all-cause hospitalizations in the treatment group from 38.8% to 30.0%, DM^*^-related hospitalizations in the treatment group decreased significantly from 35.3% to 26.9%. For site B, the treatment group showed a significant decrease in all-cause hospitalizations from 60.3% to 40.7%, DM-related hospitalizations in the treatment group decreased significantly from 54.6% to 37.0%. Care coordinator—initiated primary care clinic visits significantly decreased in the treatment group from 59.0% to 21.0%.	The apparent increase in ED^*^ visits, as seen in the results, is a result of unintended consequences in the inclusion criteria. The treatment group enrolled more patients with multiple hospitalizations, while the comparison group had a higher proportion with multiple ED visits to meet criteria.	N/A
8	The aim of this study was to investigate the impact of one-way mobile phone-based video messages on diabetes self-care, specifically assessing their effect on hemoglobin A1c (A1C) levels and SMBG^*^.	Diabetes	Mobile Phone-Based Video Messages	Offering broad lifestyle support to individuals not meeting glycemic targets despite receiving specialized diabetes care.	Both groups experienced declines in A1C Participants receiving messages experienced a greater A1C decline compared to those receiving usual care, with a 0.2% difference over 12 months (*p* = 0.002). Those who received video messages and viewed >10 per month had the greatest decline (0.6%, *p* < 0.001). Self-monitoring of blood glucose metrics showed no association with the intervention.	Hypoglycemia (<70 mg/dL) was slightly more frequent for the video messages group (*p* = 0.05 for both time ranges).	Utilizing mobile phone-based video messages for diabetes self-care significantly improves A1C levels. Technology engagement plays a crucial role in its success, and the intervention is both easy to implement and sustain.
9	Implementing endocrine triage with teleendo visits for COVID-19 patients, follow-ups, individuals above 65 years of age, and cases where a clinical assessment by an endocrinologist is not deemed absolutely necessary to achieve these objectives: mitigating the spread of the virus and preventing visit cancellations by patients. This approach is particularly crucial for fostering better adherence to the treatment process in chronic endocrine conditions.	Endocrinology issues	Phone and email (teleendo)	Developing a tailored emergency plan for outpatients with endocrine diseases, specifically catering to COVID-19-infected, elderly, frail individuals, or those with stable diseases. The goal is to limit viral spread and enable ongoing follow-up care for this patient group.	Following endocrine triage, cancellations decreased to 9%, significantly lower than the 37% patient-initiated cancellations (*p* < 0.001). During lockdown weeks, patient cancellations reduced from 47% to 19% (*p* = 0.032). Of those contacted through endocrine triage, 86% received clinical responses through face-to-face and teleendo visits. Prioritizing face-to-face visits for young patients and teleendo for 63% of geriatric patients resulted in comparable outcomes for both age groups.	N/A	The teleendo emergency plan adheres to WHO^*^ recommendations for limiting viral spread and proves beneficial for ongoing follow-up of outpatients with endocrine diseases.
10	To implement telemedicine in the pediatric endocrinology clinic during the COVID-19 pandemic, aiming to prevent the spread of the virus.	Pediatric endocrinology	E-mail and e-message	To continue caring for children with endocrine disorders and to educate patients and their parents.	Reduced hospital visits and physical contact, minimizing the risk of infection. Patients received prescription support and continued education without complications. This approach eliminated the need for workplace permissions, facilitated convenient lab tests, and prevented treatment interruptions. Closer monitoring was achieved for specific patients, and follow-up plans were effectively communicated through written messages, enhancing safety and accessibility during the pandemic.	Difficulties for illiterate patients, internet requirements, and limitations in charging. The inability to document records and occasional patient load issues were drawbacks. Addressing these challenges is crucial for effective implementation.	The most frequent diagnosis was diabetes mellitus, followed by thyroid diseases, puberty disorders, pituitary diseases, adrenal diseases, disorders of sexual development, obesity, metabolic bone diseases and so on.
11	To increase access to specialist care for people with diabetes, especially those living in rural areas. The clinic also helps improve glycemic control, treatment adherence, self-management, and clinical outcomes	Diabetes	Connected devices, remote lifestyle coaching, clinical support with a mobile app, and live video consultations with board-certified endocrinologists	To support diabetes management between office visits in the primary care.	The study reported a significant decrease in HbA1c levels across the baseline categories of >9.0%, 8.0%−9.0%, and 7.0% to <8.0%, respectively (all *p* < 0.001). Within these categories, HbA1c improved in 91.9%, 77.3%, and 63.5% of participants, respectively.	N/A	The Onduo VDC^*^ has the potential to support people with type 2 diabetes and their clinicians in managing diabetes between office visits.
12	To evaluate the effect of a telehealth system on glycemic control in children and adolescents with type 1 diabetes. The study aimed to test the hypothesis that the telehealth system would improve blood glucose control and promote better self-care compared to standard care.	Diabetes	WhatsApp, Phone, short message service	To examine the impact of a telehealth system on diabetes management.	Patients who frequently engaged with the diabetes team through telehealth experienced lower HbA1c levels after six months, demonstrating a significant improvement in diabetes control. (*p* < 0.001)	N/A	The study found that the diabetes team is most frequently contacted through WhatsApp, with 57.3% via internet messaging and 23.2% via phone. Mothers of diabetic children are the primary communicators, representing 64.6% of contacts.
13	To improve access to care for chronic diseases, especially diabetes, while minimizing the risk of contamination.	Diabetes	Telephone interview, WhatsApp (teleconsultation)	To evaluate glycemic control in type 2 diabetic patients throughout the COVID-19 pandemic, comparing their glycemic and degenerative profiles before, during, and after lockdown. Additionally, we seek to assess the effectiveness and constraints of teleconsultation as an alternative follow-up method for type 2 diabetic patients.	The mean HbA1c level during lockdown was 8.4%, slightly lower than the prelockdown level of 8.5%, indicating some success in glycemic control. Additionally, drug therapy adjustments, including insulin initiation and dose intensification, were implemented in a significant proportion of patients. The series showed a low death rate of 1.8%, and degenerative complications, while present, were relatively limited.	Slightly increase in micro and macro vascular complications such as Ischemic heart disease, cerebrovascular accident, Diabetic nephropathy, Diabetic feet. (Degenerative complications in our patients may result from both the natural course of diabetes and confinement-related factors like a sedentary lifestyle, impacting glycemic control).	N/A
14	The study focused on short-term glycemic control (HbA1c levels), medication usage for hyperglycemia, and clinical process metrics like appointment scheduling time and frequency of follow-up appointments.	Diabetes	Endocrinology eConsult a factory-calibrated glucose sensor, a web-based software.	To enhance access to endocrinology services for diabetes patients, a clinical pilot program was initiated, incorporating a revised diabetes care pathway that integrates primary care clinicians, clinical pharmacists, and endocrinologists through an eConsult model.	There were no differences in patient acceptability between CGM-enhanced eConsult, endocrinology referral, or pharmacy care. Glycemic outcomes at three months were comparable: HbA1c reduction was 1% ± 2% in endocrinology, 1.5% ± 1.1% with CGM-enhanced eConsult, and 1.6% ± 1.8% in clinical pharmacy (*p* = 0.19). Time to an initial diabetes visit with a pharmacist was significantly shorter (20 days, IQR 26) than with endocrinology (45 days, IQR 54) (*p* = 0.0001).	N/A	the use of CGM-enhanced eConsult provided patients with prompt access to endocrinology expertise, gained patient acceptance, and yielded comparable short-term glycemic outcomes to in-person consultations.
15	To enhance access to specialist care for individuals with diabetes residing in remote and rural areas through the utilization of telemedicine.	Diabetes	Videoconferencing for remote consultation	To investigate the utilization of videoconferencing for remote consultations with individuals diagnosed with diabetes.	Video consultations effectively substituted for a large proportion of in-person specialist consultations for people with diabetes. In 66% of consultations, a nurse accompanied the patient, facilitating comprehensive care. Recommendations were made in a significant percentage of consultations, including laboratory tests (75%), insulin dose adjustments (39%), and referrals to allied health professionals (13%).	For 12 patients, specialists indicated the need for a physical examination that was not possible remotely. In 34% of cases, specialists believed that a better decision could have been made with an in-person consultation. Face-to-face visits were requested for three patients, highlighting situations where video consultation had limitations.	Video consultations effectively replace in-person specialist visits for people with diabetes, demonstrating that physical examination is not a significant limitation.
16	The goal of the study is to compare how well videoconferencing consultations work compared to in-person consultations. (Evaluating how reliable it is to use remote consultation for diabetic patients.)	Diabetes	Videoconference	Diabetes evaluation and administration (alter in patients medications and recognizing diabetes complications)	The outcomes of medication change: Major change in initiation , cessation , type and injection frequency of insulin and other drugs. Minor change in dose adjustment of insulin and other drugs. Also this study proved the safety and dependability of using videoconferencing for telemedicine to manage diabetes especially for country residents.	N/A	This study is the first one that investigates whether video calls are safe for health care consultations about diabetes.
17	To check if the remote diagnosis imaging model can accurately detect and identify macular degeneration as a screening tool in clinics.	Diabetes	Remote Diagnosis Imaging Model (teleophthalmic method)	To diagnose referable macular degenerations (as a screening tool)	Remote diagnosis showed high diagnostic accuracy in identifying referable macular degeneration. Both special tests called OCT^*^ and CFP^*^ had a 94% chance of correctly detecting the condition in remote diagnosis. A nonvalidated patient satisfaction survey indicated that 76.7% of participants preferred remote imaging over standard care examination.	N/A	This information could help give treatments at the right time and may suggest a need for more research on other measurements.
18	This study wanted to find out what factors influenced diabetic patients to use telemedicine for their specialty care during the COVID-19 pandemic.	Diabetes	Telemedicine visits which conducted virtually using internet-based video applications on patients’ smartphones or personal computers.	To found the elements affected on diabetic patients’ decision to avoid using telemedicine.	Older age (50 and above) and non-English speakers were less likely to use telemedicine for diabetes management. Public insurance holders also had lower odds of telemedicine use. Reasons for choosing in-person visits included a belief in higher quality care and unfamiliarity with technology or lack of smartphone access. The main barrier was unfamiliarity with technology or lack of a smartphone, cited by 36.8% of adult patients and 20% of parents. While 53.3% of parents with pediatric patients expressed willingness to use telemedicine in the future, the majority of adult patients (81.6%) were unlikely to use it for diabetes care.	N/A	During the COVID-19 pandemic, there have been differences in the use of telemedicine for patients with diabetes based on their age, language, and insurance coverage.
19	Solving the problem of limited access to subspecialty medical care for children in rural communities.	Endocrine disease	Electronic health record	To evaluate visit attendance rates for telemedicine and in-person encounters in children receiving subspecialty endocrinology care.	Telemedicine encounters showed significantly higher attendance than in-person encounters (OR = 2.55, *p* < 0.001). However, attendance was lower for patients with nonprivate insurance or living more than 60 miles from the UCDCH in Sacramento (*p* = 0.002 for 61–100 miles, *p* < 0.001 for >100 miles).	N/A	The onset of the COVID-19 pandemic has led to a significant increase in telehealth visits, enhancing access to care for high-risk patients. Telemedicine visit attendance was found to decrease with increasing patient age, with infants having the highest rates (*p* < 0.001). Additionally, visits for obesity had lower attendance compared to those for diabetes (*p* < 0.001).
20	The goal of this study was to see if using a smart phone app that promotes a healthy lifestyle can change how well people do in their treatment an endocrinology outpatient clinic.	Diabetes	Smart phone application(Sick Kick Health)	To modify the treatment outcomes at an endocrinology outpatient clinic.	In the intervention group unlike the control group, there was a statistically significant reduction in HbA1c level (61 ± 21.4 to 52.7 ± 15.2 mmol/mol), diabetes distress (19.5 ± 16.5 to 11.7 ± 13.4), and anxiety symptoms (from 5.4 ± 4.0 to 4.1 ± 3.8)	N/A	N/A
21	The objective of this study was to determine the impact of the MyT1DHero program on the well-being of adolescents with diabetes. Our intention was to determine if the program could enhance their blood sugar levels, how effectively they adhere to their diabetes care, and the level of satisfaction they have with the program.	Diabetes	Mobile app MyT1DHero	To improve diabetes outcomes in adolescents, specifically the hemoglobin A1c (HbA1c) levels, diabetes care adherence, and quality of life and to gather data on satisfaction rate with this intervention	-Demonstrated a significant link between app usage (high/medium/low) and improved HbA1c levels (F1,20 = 9.74, *p* < 0.005; R2 = 0.33), indicating that higher usage of MyT1DHero led to greater improvements in adolescents’ HbA1c levels.-Significant improvements were found in the diabetes behavior and quality of life of the adolescents.-The parents’ perceptions of diabetes behavior also improved from before the study (mean 4.04 [SD 0.50]) to after the study (mean 4.34 [SD 0.47])-there was an improvement in the family conflict for the adolescents from before the intervention (mean 2.45 [SD 0.55]) to after the intervention (mean 2.61 [SD 0.45]) -The scores of the child and the parent Revised Diabetes Family Conflict Scale were significantly correlated. -Both the adolescents (mean 2.19 [SD 0.94]) and the parents (mean 2.26 [SD 1.29]) rated the intervention as satisfactory. -The greater the number of blood glucose readings and messages adolescents logged, the more improvement was observed in their HbA1c levels.	-No significant decrease in HbA1c levels (8.94 [SD 1.46] to 8.87 [SD 1.29])-the results of the parents’ perception of conflict did not change significantly	A mobile app intervention, MyT1DHero, aims to enhance positive communication between parents and adolescents with T1D, utilizing separate app interfaces for each. The intervention is designed to improve diabetes-related outcomes through enhanced communication about T1D management.
22	To compare Teleophthalmology with in-person ophthalmology	DR (diabetic retinopathy) (165 eyes of 95 people had DR	Ultra-widefield fundus camera to teleopathalmology	DR screening	Across the world, teleophthalmology is widely employed to enhance accessibility to eye care and assist physicians in prioritizing individuals requiring specialized treatment. The research has found that patients are satisfied with telemedicine as a way to receive health care and also has proven that teleophthalmology is as effective as the traditional system in achieving the desired clinical outcomes. These eyes benefited from prompt management to prevent retinopathy progression, as patients might have been unaware of diabetes affecting their eyes, and their vision may not have been significantly affected.	The secondary result measure of poor picture securing was seen in one person who had an picture acquired in one eye that might not be evaluated due to awful picture quality. This implies that picture acquisition with UWF^*^ camera by a prepared nursing faculty is doable	Less than 12% of eyes required additional tests and treatment. Consequently, immediate measures were taken to prevent further deterioration of retinopathy by effectively managing these eyes. The benefit is made clear
23	The study consider assessed whether an intervention utilizing the SMS* by individual cellular phone and web would progress the levels of plasma glucose of obese type 2 diabetes at 3, 6, 9, and 12 months and to decrease the body weight	Diabetes	Personal cellular phones or computer internet	Collect data and analyze the process of changes in HbA1c and 2HPPT in the intervention group vs the control group.	HbA1c decreased 1.22% at 3 months, 1.09% at 6 months, 1.47% at 9 months, and 1.49% at 12 months in the intervention group (*p* < 0.05) The intervention group had a decrease of 2-h postprandial test (2HPPT) of 120.1 mg/dL at 3 months, 58.9 mg/dL at 6 months, 62.0 mg/dL at 9 months, and 102.9 mg/dL at 12 months (*p* < 0.05) One main benefit of this service is that patients can easily reach out to researchers whenever they need to and benefited a plan based on their specific personalities.	N/A	Most type 2 diabetic patients in Korea are not overweight, which is different from Caucasians but similar to other Asian countries. Many online programs for managing diabetes on the internet have developed with the aim of providing emotional support and giving patients more information to help them take care of themselves and receive counseling. This study found evidence that online services can work just as well as in-person guidance and treatment for managing diabetes.
24	To utilize telenursing for patients’ self-monitoring, self-management to attain glucose targets, and prevent complications	Diabetes	Telephone (-information about diabetes self-monitoring and self-care, diet, exercise, optimal glucose targets and insulin self-titration.-via USB connected to the glucose meter, via e-mail or telephone calls.-y received telephone calls every Thursday	Investigating the level of blood glucose control and its changes and encouraging diabetic patients to self-monitoring.	Both groups had similar demographics and baseline characteristics. In the intervention group, there was a significant decrease in blood glucose levels across various measurements (morning, preprandial, postprandial), as well as a significant reduction in HbA1c over time (8.3% at the start to 7.8% at the end of the study, *p* = 0.03). Additionally, the intervention group had fewer omitted glucose measurements compared to the control group.	N/A	In this research, using phones is an affordable and efficient way to provide good health care to patients, particularly those who do not have easy access to health care providers. In addition, it can be used in any country (whether it is developing or developed) and with patients who have a low social and economic standing.
25	The aim of this study is to manage and monitoring diabetes patients via a web-based diabetic patient management system instead of face-to-face doctor — patient interviews in the hospital.	Diabetes	SMS	To minimize the impact on social economic and make personalized diabetes management possible	After using the management program, the average HbA1c levels improved from 7. 5 ± 15% to 70 ± 11%, (*P*= 0.003). The average levels of triglycerides and HDL^*^ cholesterol in the blood improved. The HbA1c level decreased from 8. 4 ± 12 after using this program on patients with an initial HbA1c level of 7% or higher, the average HbA1c level decreased to 7. 5 ± 10% (*p* = 0. 010) Most participants were satisfied with this web-based diabetic patient management program.	N/A	According to the survey results, it was observed that type 1 diabetic patients showed higher levels of contentment with our web-based management system because they wanted to be in touch with their health care providers more often to better manage their diabetes. for three months, participants utilized a specialized web-based diabetes management system to transmit their self-measured blood glucose levels, medication details (including dosages), meal quantities, and exercise intensity to their health care providers.
26	The study aimed to develop a mobile telecare system for automatic data transmission from patients to physicians multiple times a day and to evaluate its clinical efficiency in intensive insulin treatment of newly diagnosed type 1 diabetic patients without clinic visits.	Type 1 Diabetes	Mobile logbook	allowing for automatic transmission of the patient collected data directly to the physician multiple times a day and enabling the physician to control the patient’s self-management routine.	The mean blood glucose decreased from 7.2 ± 1.7 mmol/L to 6.1 ± 1.0 mmol/L in the third week of the study (*p* = 0.02), and the J-index decreased from 30.2 ± 19.2 to 19.7 ± 7.7 (*p* = 0.04). Hemoglobin A1c decreased from 11.8 ± 3.3% to 8.6 ± 1.2% in one month (*p* = 0.0002). The total daily insulin dose declined from 39.9 ± 8.5U to 20.0 ± 9.6U (*p* = 0.000006). The number of hypoglycemia episodes per patient per day decreased by 66% (*p* = 0.08), and the number of hyperglycemia episodes was reduced by 47% (*p* < 0.0001). The duration of treatment supported by the TeleMed system significantly positively influenced the MBG^*^ and J indices, as well as the daily number of hyperglycemic episodes.	N/A	N/A
27	The goal of this study was to understand how teleconsultation and in-person consultation affect glycemic control for diabetic veterans. We hypothesized that the level of control would be the same for patients who received care through telehealth as compared to those who had in-person visits.	Diabetes	Mobile health technology	For various purposes such as communication between doctors and patients, diagnosis, providing care, educating patients, sharing information, monitoring health, and sending reminders.	HbA1c significantly improved from baseline in both TH and FTF^*^ groups, with no significant difference between them (*p* = 0.24). Additionally, TH visits were associated with a potential savings in median travel distance of 231.2 miles per trip (equivalent to $94.79 saved per trip).	N/A	Although TH* (telehealth) care is gaining popularity among an expanding user base, yet significant obstacles continue to impede its ease of use. These problems include differences in how much insurance will pay for TH in different states, being able to practice medicine in different states, and keeping medical information private.
28	To explain the clinical experience of utilizing digital technology to monitor and help obese patients. Additionally to see how these patients’ weight changed when they received medical assistance via telemedicine.	Obesity	Teleconsultation	To manage and observe how obese patients’ weights change.	We noticed that people followed the program well and lost an average of 4. 1 Kg after three months. This weight loss was still maintained after six months. Overall, telemedicine and other digital health strategies can be beneficial for both patients and health care providers. They can help ensure that patients receive consistent care, and they have been found to be effective in helping obese patients.	The only groups of patients that didn’t have a significant impact were those who were over 60 years old and those who were classified as (adiposity-based chronic disease ) ABCD stage 2.	Johnson and his team conducted a study where they compared a weight loss program done through video calls with one done in person. One main difference between our study and Johnson’s study is that we didn’t have tools for health care providers to track progress. This might be why the patients in our study didn’t lose as much weight as those in Johnson’s study.
29	The study wants to prove that TeKnO T1D can improve endocrinology fellows’ knowledge about insulin pumps and CGM use in the management of pediatric type 1 diabetes	Diabetes	app and Email (TeKnO T1D, an online, case-based, spaced education curriculum)	To gather information on how children with type 1 diabetes use insulin pumps and CGMs to manage their condition.	The pump group had increased from 35.0 ± 15% on the pretest to 61.1 ± 17% on the posttest, a 12.2% greater improvement on pump specific items than the CGM group (*p* = 0.03). The CGM group had increased from 30.3 ± 15% on the pretest to 61.4 ± 21% on the posttest, a 28.7% greater improvement on CGM-specific items than the pump group (*p* < 0.001) Before completing the curriculum, 25% of learners in the pump group felt they had a sufficient understanding of insulin pumps, compared to 66.7% on the posttest questionnaire.	N/A	TeKnO T1D demonstrated its effectiveness in enhancing endocrinology fellows’ knowledge and confidence in utilizing insulin pumps and CGM for managing pediatric type 1 diabetes.
30	Identification of factors to increase efficacy of telemedicine screening for DR	Diabetes	Fundus photography	Telemedicine screening	N/A	N/A	Screening in areas with a higher prevalence of diabetes resulted in a higher prevalence of DR being detected.
31	Type 2 Diabetes Management	Diabetes	N/A	Endocrinology electronic consultation	Intervention group had a 0.89 (SD 1.45) decrease in HbA1c. There was a 19.3% increase in patients prescribed GLP-1 RA or SGLT2i in the intervention group. There were also significant increases in prescription rates of metformin (3.1% vs −3.1%, *p* = 0.03) and sulfonylureas (1.5% vs −6.9%, *p* = 0.03).	N/A	Unsolicited eConsultation was associated with increased prescribing of glucose-lowering medications without significant difference in HbA1c.
32	Reduction of coronary heart disease	Diabetes	Printed materials and computers, SMS and phone calls	Education	The average blood sugar measurements in the control and intervention groups were higher compared to follow-up in both groups.	The change in treatment in the control group (33.3%) was relatively high compared to the intervention group (11.8%).	Mobile education has great potential to improve patient care and increase social interactions and lifestyle enhancement.
33	To reduce stress among patients with diabetes	Diabetes	Mobile Phone	Education and counseling	The average stress scores for the control and intervention groups were similar (18.9). At the 3-month follow-up, these scores decreased to 17.05 in the intervention group, while they increased to 20.7 in the control group.	N/A	Intervention in the form of intensive lifestyle training and phone calls and text messages reduces the level of stress in diabetics.
34	For Promotion of Physical Activity Among Newly Diagnosed Patients of Type II Diabetes	Diabetes	Mobile application	N/A	A significant decrease in weight, BMI, waist circumference, hip circumference, body fat percentage and SBP^*^ was observed in the intervention group.	N/A	Mobile applications are affordable and clinical exercises are helpful in promoting physical activity.
35	Assessing patient and provider perspectives for the use of telemedicine	Endocrinology, nephrology, orthopedic surgery, and rheumatology	Video visit , Makoul CAT and RAND VSQ925 via REDCap	Data collection	In patients, 72.7% believed that they had completed some components of a clinical examination. The average overall satisfaction was 86.7 ± 19.3%.	N/A	N/A
36	Self-management in Patients with diabetes	Diabetes	Mobile Phone	Data collection	Self-management related to diet planning is (96%), and checking blood sugar is (90.9%).	N/A	Most patients with type 2 diabetes tend to use mobile phones and the internet for telemedicine.
37	Self-management	Diabetes	Mobile Phone	Education and data collection	Patients of the intervention group showed a significant increase from 17.3% in the beginning to 43.6% in the end, and the control group showed a significant increase from 13.6% in the beginning to 15.9%.	N/A	Mobile phone technology was beneficial in reducing HbA1c levels in diabetic patients in the intervention group. Also, reducing LDL and following a diabetic diet plan can reduce HbA1c in these patients.
38	Self-management	Diabetes	Mobile SMS	Education	At first, no significant difference was observed in terms of the level of knowledge and beliefs. But after the SMS intervention, the level of awareness and beliefs was significantly higher in the SMS group without SMS.	N/A	Education through SMS is an important variable that increases the knowledge and belief of subjects.
39	Self-management	Diabetes	REDCap	Education	Patients tended to decrease mean HbA1c and glucose variability and nonsignificant increase in hypoglycemic episodes.	N/A	Diabetes care delivered via telemedicine was associated with time and cost savings, high rates of appointment adherence, and high patient satisfaction.
40	Self-management	Diabetes	Welltang application	Education	The average reduction in HbA1c was 1.95% in the intervention group and 0.79% in the control group. In general, self-care behaviors improved in the patients of the intervention group.	N/A	N/A
41	Using telemedicine with the help of nonmydriatic fundus photography in DR screening	Diabetes	NMFP* and FFA*	To explore the efficacy of NMFP-enhanced telemedicine in assessing DR and its various stages.	Accurate method for examining the fundus, especially suitable for endocrinology inpatient care and primary health care for diabetic patients.	N/A	This approach simplifies patient assessments and significantly improves clinical results in DR management. The new theories proposed by this study include the importance of integrating NMFP-assisted telemedicine into endocrinology and primary health care for enhanced DR management.
42	To investigate the utility of point of care screening of DR* and the impact of a telemedicine program to overcome current challenges.	Diabetes	Single-field nonmydriatic fundus photography	Screening patients using the single-field nonmydriatic fundus photography obtained with a retinal camera	To provide the opportunity for early detection of patients with sight threatening retinopathy.	N/A	N/A

CFP, color fundus Photography; CGM, continuous glucose monitoring; DM, diabetes mellitus; DR, diabetic retinopathy; ED, emergency department; FFA, fundus fluorescein angiography; FTF, face-to-face; HDL, high-density lipoprotein; IP, in-person; MBG, mean blood glucose; NMFP, nonmydriatic fundus photography; OCT, optical coherence tomography; PCOS, polycystic ovary syndrome; PCPs, primary care providers; PE, physical examination; REI, reproductive endocrinology and infertility; SBP, systolic blood pressure; SMBG, self-monitoring of blood glucose; SMS, short message service; TH, telehealth; TIR, time in range; UWF, ultra-widefield fundus; VDC, virtual diabetes clinic; WHO, world health organization.

### Outcomes of the interventions

Our review elucidates the primary objectives of telemedicine interventions in managing chronic endocrine conditions. These interventions effectively address issues such as access barriers,^24,25,43,49,54,58^ timely monitoring,^20,38,44,50,57,59,60^ and glycemic control in diabetes^29,31,39,42,45,48,50,59^ Most important, they also enhance patient education and self-management of their disease, provide lifestyle advice,^23,32,34–37,41,47^,^51^,^52^,^55^,^56^,^61^ and contribute to the reduction of costly complications.^27,35,40,51^ Notably, these interventions have proven particularly beneficial during the COVID-19 pandemic, demonstrating the adaptability and resilience of telemedicine in times of viral outbreaks.^17,38,57^

The positive outcomes of telemedicine interventions were manifold and significant. These interventions led to high levels of patient satisfaction,^24,25,45^ reduced HbA1c levels,^20,23,48–50,52,56,58^ improved self-care behaviors,^32,35,38,41,44,47,50,52,55^ and decreased glucose variability.^42,51,60,61^ Also, some studies showed it lowered the complications and hospitalizations in patients.^23,53^ Telemedicine was particularly effective during the COVID-19 pandemic, reducing infection risk^38,45^ and facilitating health care access.^25,29,43,45,57^ For instance, one study reported a significant decrease in HbA1c levels from 8.3% at the start to 7.8% at the end of the study (*p* = 0.03).^51^ This demonstrates the effectiveness of telemedicine in improving glycemic control in diabetes patients. Another study found that telemedicine encounters showed significantly higher attendance than in-person encounters, particularly for patients living more than 60 miles from the health care facility.^54^ While telemedicine has its benefits, studies highlight certain drawbacks. These include the need for technical proficiency,^38,45^ perceived lower care quality,^24,25,45^ potential privacy risks, and higher costs compared to traditional clinics.^45^ Some patients preferred in-person visits, and telehealth physical exams presented challenges, with specialists occasionally requiring physical examinations not possible remotely.^26,43^ Certain studies reported no significant decrease in HbA1c levels^50^ and a slight increase in micro- and macrovascular complications such as ischemic heart disease, cerebrovascular accidents, and diabetic nephropathy.^58^ These findings contribute to a nuanced understanding of the multifaceted impacts of telemedicine interventions, informing the global discourse on their efficacy and limitations.

## Discussion

The recent decades have witnessed significant technological advancements, the implications of which are increasingly being realized in the medical sector. The incorporation of these advancements into medical practice has transformed caregiving, introducing a new paradigm in patient care. Telehealth applications, in particular, have found extensive use in various medical fields, including endocrinology, serving as a platform for remote patient monitoring, consultation, disease management, and medication adjustment. As of 2019, 76% of U.S. hospitals had adopted some form of telehealth modalities.^62^

The onset of the COVID-19 pandemic presented unprecedented challenges in health care delivery, necessitating the adoption of innovative solutions to optimize and ensure care delivery. This led to an accelerated integration, utilization, and development of telehealth, which can address traditional caregiving difficulties and problems.^63^ Numerous studies have highlighted the effectiveness of telehealth solely or in conjunction with traditional health care, particularly during the COVID-19 pandemic, while also acknowledging its limitations.^64–66^

Telehealth has found applications in a wide range of medical fields, including cardiovascular diseases, pulmonary diseases, dermatology, nephrology, psychiatry/psychology, neurology, multidisciplinary care,^67^ cancer treatment,^68^ and pediatric care.^69^ Endocrinologists had adopted telehealth as a standard care modality even before it became mainstream in other medical fields and before the COVID-19 pandemic.^70^ This can be attributed to the chronic nature of endocrine disorders, which primarily involve patient history taking, laboratory, and imaging results, rather than physical examination. Moreover, endocrine diseases necessitate long-term follow-ups, which can be conveniently facilitated through telehealth.

Telehealth offers several benefits, such as improved access to health care services, especially for patients in remote areas with a shortage of health care professionals^7^ and elderly people.^71^ It enhances patient satisfaction by saving time and eliminating unnecessary travel, thereby reducing associated costs. Telehealth also mitigates the risk of infections acquired from health care facilities.^72^ The user-friendly nature of telehealth can lead to greater satisfaction,^45^ acceptability, and feasibility,^73^ resulting in higher attendance than in-person visits and a higher visit completion rate^.19^ Furthermore, telehealth has the potential to improve health outcomes by enabling timely intervention and promoting patient engagement in self-care and adherence to therapy.^74^ It also serves as an effective medium for delivering health education,^75^ physical activity, and diet monitoring.^76^

However, the implementation of telehealth in endocrinology is not devoid of challenges. Successful execution of telehealth requires resources and extensive infrastructures allocated to health care systems by administrations and governments.^65^ This increases technology-related costs^77^ which may not be feasible, especially in low- and middle-income countries. Technical issues, such as poor internet connectivity^78^ and lack of hardware equipment such as smartphones, wearables, and laptops, can impede care delivery.^65^ Additionally, the absence of physical interaction may limit the clinician’s ability to conduct comprehensive assessments^.26^ There are also concerns about the quality of care delivered via telehealth, with some patients and providers preferring traditional in-person visits due to technology illiteracy^79^ or the inability to reach treatment goals.^66^ Moreover, with the advancement of electronic health records and large databases, patient data privacy has become a contentious issue, necessitating the adoption of regulatory parameters. Insurance reimbursement considerations can also pose a barrier to the effective implementation of telehealth.^77^

In the realm of telehealth and endocrinology, our systematic review presents a novel approach by encompassing all endocrine conditions, rather than focusing solely on specific diseases. This comprehensive scope allows for a more holistic understanding of the implications of telehealth in endocrinology, thereby filling a significant gap in the existing literature. Our findings largely align with previous research, underscoring the benefits of telehealth such as increased accessibility, cost-effectiveness, and patient satisfaction. However, we also acknowledge the limitations, including technological barriers, data security concerns, and the potential for reduced interpersonal interaction. Our review suggests that the advantages of telehealth in endocrinology significantly outweigh the drawbacks, reinforcing its potential as a transformative tool in health care delivery.

Despite these challenges, the future of telehealth in endocrinology appears promising. With advancements in technology and growing acceptance among patients and providers, telehealth is poised to become an integral part of endocrine care. Further research is warranted to develop more robust evidence to establish guidelines for its use and to explore its potential in managing endocrine disorders in hard-to-reach populations. From a policy perspective, it is imperative to create a conducive environment for the adoption of telehealth. This includes addressing regulatory issues, reimbursement policies, and ensuring the privacy and security of patient information.

### Limitations

In this study, we studied the available recent resources on telehealth applications in endocrinology. Considering the broadened telehealth field, many studies did not differentiate between the different technologies used, and we had to perform a very careful screening to include more relevant studies. Also, some of the included studies did not clearly address the pros and cons of telehealth technologies for endocrinology.

## Conclusions

In conclusions, telehealth holds significant potential in transforming endocrine care. While there are challenges to its implementation, the benefits it offers underscore its value as a health care delivery model. In the right context, such as appropriate diseases that can benefit from telehealth implementation and suitable infrastructures, it can be beneficial. In the coming years, with deeper integration of electronic medical records with telehealth systems and technological advancements such as high-speed connections such as 5G internet, which comes at lower costs, telehealth is projected to mature and surpass in-person encounters in terms of attendance. As we continue to navigate the digital health landscape, it is crucial to leverage telehealth solutions to enhance the quality of care in endocrinology.

## Data Availability

The authors stated that all information provided in this article could be shared.

## Abbreviations Used

CIs: Confidence Intervals
COVID-19: Coronavirus disease 2019
HbA1c: Hemoglobin A1C
NOS: Newcastle-Ottawa Scale
PRISMA: Preferred Reporting Items for Systematic Reviews and Meta-Analyses
RCT: Randomized Controlled Trial
SD: Standard Deviation
SMS: Short message service
